# Histopathologically TMA-like distribution of multiple organ thromboses following the initial dose of the BNT162b2 mRNA vaccine (Comirnaty, Pfizer/BioNTech): an autopsy case report

**DOI:** 10.1186/s12959-022-00418-7

**Published:** 2022-10-06

**Authors:** Ryo Kaimori, Haruto Nishida, Tomohisa Uchida, Mari Tamura, Kohji Kuroki, Kumi Murata, Kinta Hatakeyama, Yoshihiko Ikeda, Kisaki Amemiya, Akira Nishizono, Tsutomu Daa, Shinjiro Mori

**Affiliations:** 1 Department of Forensic Medicine, Faculty of Medicine, Oita University, 1-1 Idaigaoka, Hasama-machi, Yufu, Oita 879-5593 Japan; 2 Department of Diagnostic Pathology, Faculty of Medicine, Oita University, 1-1 Idaigaoka, Hasama-machi, Yufu, Oita 879-5593 Japan; 3 Department of Microbiology, Faculty of Medicine, Oita University, 1-1 Idaigaoka, Hasama-machi, Yufu, Oita 879-5593 Japan; 4 Department of Pathology, National Cerebral and Cardiovascular Center, 6-1 Kishibe-Shimmachi, Suita, Osaka 564-8565 Japan; 5 Research Center for GLOBAL and LOCAL Infectious Diseases, Faculty of Medicine, Oita University, 1-1 Idaigaoka, Hasama-machi, Yufu, Oita 879-5593 Japan

**Keywords:** SARS-CoV-2, Coronavirus disease 2019, Vaccination, BNT162b2, Thrombotic microangiopathy, Thrombosis

## Abstract

**Background:**

Coronavirus disease 2019 (COVID-19) has spread worldwide. Vaccination is now recommended as one of the effective countermeasures to control the pandemic or prevent the worsening of symptoms. However, its adverse effects have been attracting attention. Here, we report an autopsy case of multiple thromboses after receiving the first dose of the BNT162b2 mRNA vaccine (Comirnaty, Pfizer/BioNTech) in an elderly woman.

**Case presentation:**

A 72-year-old woman with a history of diffuse large B-cell lymphoma in the stomach and hyperthyroidism received the first dose of the BNT162b2 mRNA vaccine and died 2 days later. The autopsy revealed multiple microthrombi in the heart, brain, liver, kidneys, and adrenal glands. The thrombi were CD61 and CD42b positive and were located in the blood vessels primarily in the pericardial aspect of the myocardium and subcapsular region of the adrenal glands; their diameters were approximately 5–40 μm. Macroscopically, a characteristic myocardial haemorrhage was observed, and the histopathology of the characteristic thrombus distribution, which differed from that of haemolytic uraemic syndrome and disseminated intravascular coagulation, suggested that the underlying pathophysiology may have been similar to that of thrombotic microangiopathy (TMA).

**Conclusion:**

This is the first report on a post-mortem case of multiple thromboses after the BNT162b2 mRNA vaccine. The component thrombus and characteristic distribution of the thrombi were similar to those of TMA, which differs completely from haemolytic uraemic syndrome or disseminated intravascular coagulation, after vaccination. Although rare, it is important to consider that fatal adverse reactions may occur after vaccination and that it is vital to conduct careful follow-up.

**Supplementary Information:**

The online version contains supplementary material available at 10.1186/s12959-022-00418-7.

## Background

Severe acute respiratory syndrome coronavirus 2 (SARS-CoV-2) is a novel virus that was first reported in late 2019 as the causative agent of coronavirus disease 2019 (COVID-19), which has spread worldwide [1]. The risk of developing severe COVID-19 symptoms is particularly high among the elderly and individuals with underlying diseases [2]. One of the effective countermeasures to control the pandemic or prevent the worsening of symptoms, as dictated by the World Health Organization, is vaccination. With the rapid implementation of vaccination programmes and a subsequent increase in the total number of vaccinated individuals, adverse vaccine effects have been attracting attention. Serious side effects such as (peri)myocarditis following mRNA-based vaccination [3–5] and thrombosis following adenoviral vector-based vaccination [6–8] have been reported. Although a number of post-mortem investigations of fatalities following mRNA vaccination showed myocardial infarction or myocarditis [9], autopsy reports showing thrombotic microangiopathy (TMA)-like multiple organ thromboses following mRNA vaccination are rare. Here, we report an autopsy case of a patient who died from systemic thrombosis within 2 days of receiving the BNT162b2 mRNA vaccine (Comirnaty, Pfizer/BioNTech).

## Case presentation

A 72-year-old woman with a history of hyperthyroidism, an unspecified penicillin allergy developed in her 20s, and diffuse large B-cell lymphoma in the stomach received the first dose of the BNT162b2 mRNA vaccine at about 9 a.m. on day X. Her pre-vaccination screening questionnaire for the COVID-19 vaccine showed that her body temperature on the vaccination day was 35 °C, and no other notable findings were described. Her lymphoma showed a complete response to chemotherapy with an uneventful course, and her hyperthyroidism was under control with oral therapy. She had no medical history of deep vein thrombosis, systemic lupus erythematosus, recurrent pregnancy loss, haematuria, and haematopoietic stem cell or solid organ transplantation. Laboratory testing showed that her liver and kidney function, as well as blood count, were within normal limits one month before vaccination. The absence of thrombocytopenia and anaemia was confirmed 10 days before vaccination. She felt unwell at the vaccination venue immediately after the vaccination; however, her condition improved with some rest after which she went home. About 4 p.m. on day X + 1, she developed fatigue, nausea, chest pain, and back pain, and around 8 p.m. on day X + 1 LINE, a social networking service, showed a “Read” mark suggesting that she was alive. She was found deceased in her house on day X + 2. Life-saving procedures were not administered because rigor mortis was noted in the muscles of the jaw. A medicolegal autopsy was performed approximately 24 h after she was found deceased to investigate the manner of her death, since vaccination was suspected to have been the cause.

## Autopsy findings

**Fig. 1:**
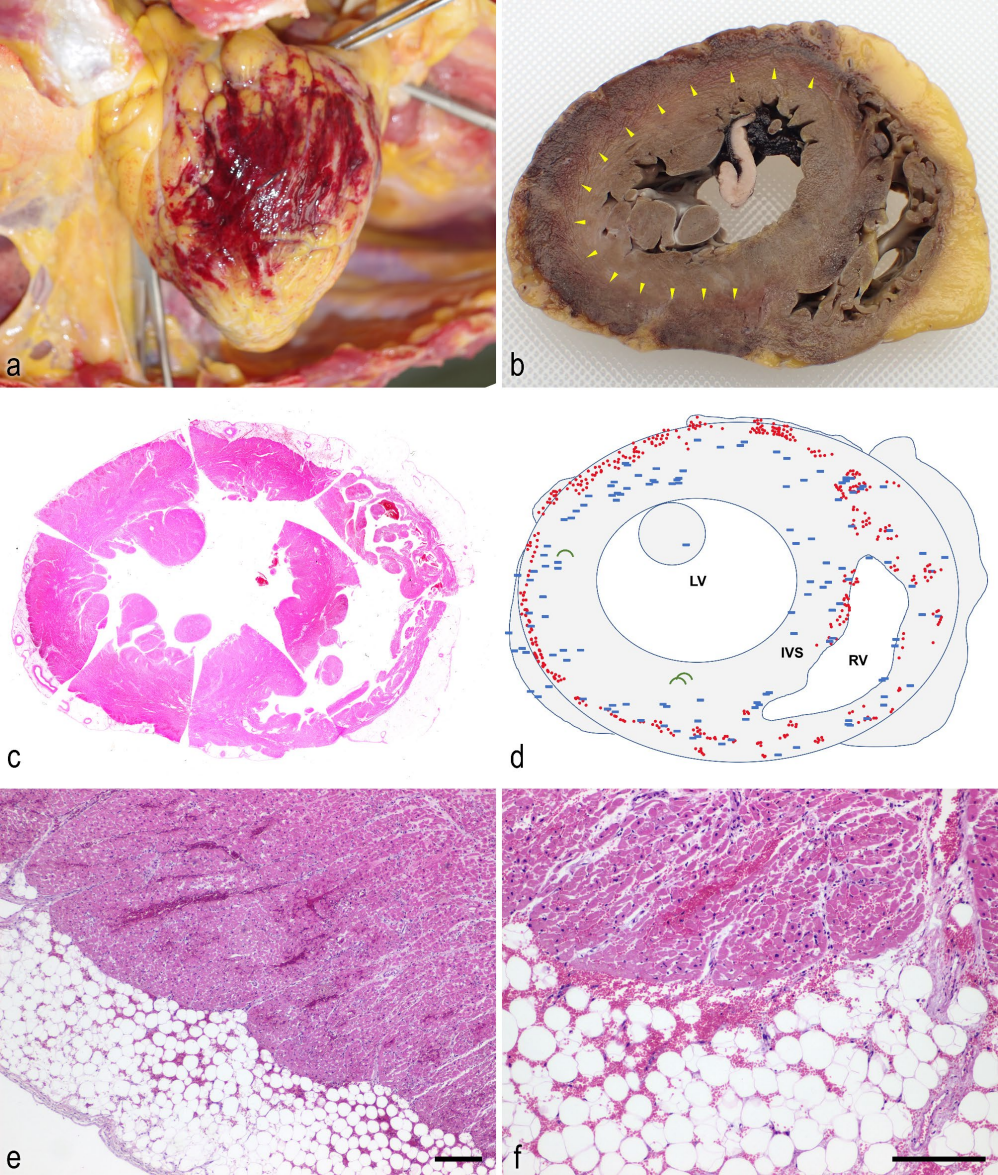
**Macroscopic and microscopic findings of the heart. a**: Macroscopic haemorrhage in the posterior pericardium in situ at the autopsy. **b**: Gross findings of the heart after fixation. The cut surface of the heart after fixation shows black-red discoloration (arrowhead) in the entire circumference of the pericardium and pericardium-side myocardium. **c**: Scanning magnifications of the heart with haematoxylin-eosin (HE) staining. **d**: Schematic illustration of the microscopic pathology. The red dots indicate the microscopic haemorrhage, the blue rectangle indicates the thrombus, and the green arc indicates the contraction band necrosis. LV; left ventricle, IVS; interventricular septum, RV; right ventricle. **e** and **f**: Low- and high-power views of the haemorrhage in the cardiomyocytes. The haemorrhages were found in the pericardium and pericardium-side myocardium, which is compatible with the discolouration in macroscopic observation. Scale bars indicate 100 μm (**e**, **f**)

At autopsy, the patient’s body length and weight were 155 cm and 53.0 kg, respectively. There were no reddening or wheals observed on the body surface. The heart weight was 394 g, and 170 mL of concentrated yellow-translucent pericardial fluid with fibrinous precipitate was present in the pericardium. Marked petechial haemorrhage was found on the surface of the posterior pericardium (Fig. 1a). Gross examination after 10% buffered formalin fixation revealed black-red discoloration throughout the circumference of the pericardium and outer surface of the myocardium (Fig. 1b). The left anterior descending artery showed only 25% angiostenosis, and no evident obstruction was noted in the coronary arteries. Marked petechial haemorrhage was also noted on the surface of the lungs, liver, kidneys, and spleen (Additional file 1a); on the diaphragm, lateral and posterior pleura; and on the mucosal surfaces of the oesophagus (Additional file 1b), stomach, and duodenum. An additional picture file shows haemorrhagic lesion in the spleen and oesophagus [see Additional file 1]. Haemorrhagic lesions were not observed in the intracranial space, and thrombosis was not noted in the superior sagittal sinus. The bladder urine was not bloody. There were no findings associated with traumatic injuries.

## Histopathological findings

**Fig. 2:**
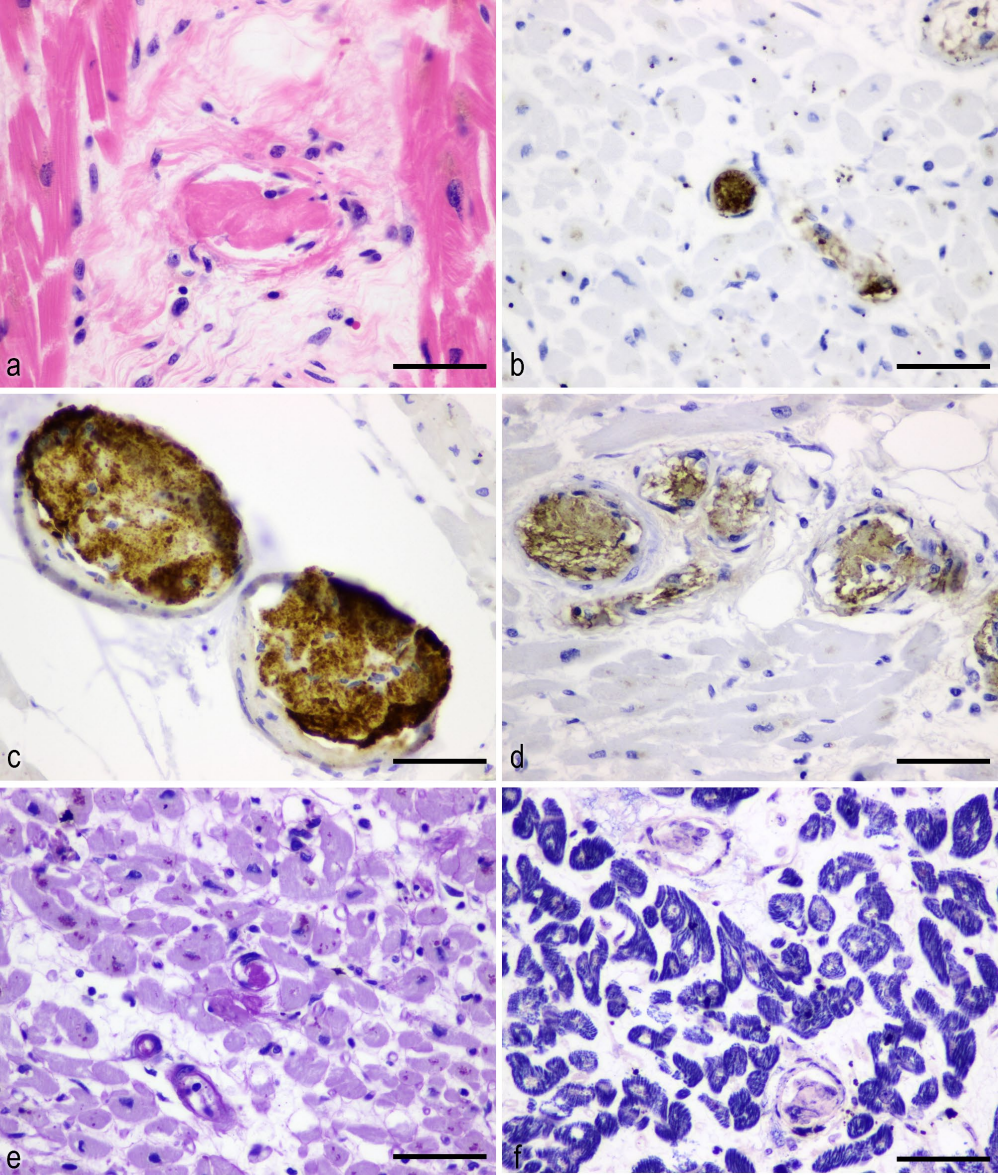
**Immunohistochemistry of microthrombi in the heart.** Microvascular hyaline thrombus stained with **a**: HE staining. **b**: anti-CD42b. **c**: anti-CD61. **d**: anti-vWF. **e**: Periodic acid Schiff staining. **f**: Phosphotungstic acid haematoxylin staining. Scale bars indicate 50 μm (**a**-**f**)

Histopathological examination revealed multifocal vacuolation and lipofuscin pigmentation in cardiomyocytes. Immunohistochemical (IHC) staining showed positivity for anti-C4d antibodies (rabbit polyclonal, catalogue no. 12-5000; American Research Products, Inc., Belmont, MA, USA; 1:400) in the vacuolated cells of the left ventricular free wall (Additional file 2a and 2b). An additional picture file shows anti-C4d -positive vacuolated cells [see Additional file 2]. Numerous microthrombi without inflammatory cells were found in the small vessels, arterioles, and capillaries of the anterior, posterior, and lateral walls of the left ventricle, right ventricle, and interventricular septum, located predominantly at the border between the haemorrhagic and non-haemorrhagic areas (Fig. 1c and f). These microthrombi were found to be immunoreactive for anti-CD42b (mouse monoclonal, clone MM2/174; Novocastra, Newcastle upon Tyne, UK; 1:100), anti-CD61 (mouse monoclonal, clone 2f2; Leica Biosystems Newcastle, Ltd., Newcastle upon Tyne, UK; 1:500), and anti-von Willebrand factor (vWF) (mouse monoclonal, clone F8/86; Dako, Carpinteria, CA, USA; 1:25) antibodies, as well as periodic acid Schiff staining (Fig. 2a and e). An additional picture file shows ultrastructural analysis of a thrombus in the heart in detail [see Additional file 3]. The vascular diameter of the microthrombi was approximately 5–40 μm, and the microvessels were congested and dilated. Increased eosinophilic cardio-myofibrillar bundles were localised, and these bundles were highlighted with Luxol Fast Blue (LFB) stain. LFB staining also demonstrated many irregular deep-blue wavy contraction bands extending across the fibres in the region of the cardiomyocytes, indicating contraction band necrosis. No significant inflammatory cell infiltration or fibrotic lesion was detected in the cardiac specimen. In addition, IHC for anti-SARS-CoV-2 spike glycoprotein (rabbit monoclonal, clone HL6; GeneTex, Inc., Irvine, CA, USA; 1:100) was negative in the cardiomyocytes.

The kidneys were found to have focal interstitial haemorrhage, as well as fibrin and platelet thrombi in the glomerular capillaries and afferent/efferent arterioles. CD61-positive thrombi were detected in the small vessels and arteriole of the adrenal subcapsular region. Although IHC staining showed positivity for anti-C4c antibodies partially in the glomerular tuft and capillaries, as well as the parenchyma of the renal tubule, it was difficult to assess. Jones sliver (periodic acid-methenamine silver) staining revealed the enlarged subendothelial space of the glomerular basement membrane in some regions of the glomerulus. Microthrombi were also found in the liver, pancreas, cerebral cortex, cerebellum, and pons; however, no ischaemic change was noted. Thrombi covered with endothelium were not detected in any of the tested organs. The vascular diameter of the vessels involved in thrombi formation in these organs was similar to that of those in the heart. In the lungs, the pulmonary capillaries were congested, and pulmonary oedema was noted. Haemorrhagic lesions were found in the capsule and parenchyma of the spleen and lateral lacunae of the superior sagittal sinus. There were no apparent findings associated with infections such as inflammatory cell infiltration or autoimmune diseases including systemic sclerosis and nephropathy.

Virus isolation tests did not detect any viruses in the pericardial fluid. Her blood was not biochemically tested for factors such as autoantibodies, a disintegrin-like and metalloproteinase with thrombospondin type 1 motifs, member 13 (ADAMTS13) activities, and platelets, because of post-mortem changes at the time of autopsy.

As mentioned above, platelet microthrombi were detected in multiple organs, predominantly in the heart, and injuries to the other organs were limited. Therefore, it was conceivable that the sudden death was cardiac in origin.

## Discussion and conclusions

Since March 2021, unusual thrombosis and thrombocytopenia have been reported after receiving the ChAdOx1 nCoV-19 vaccine, an adenoviral vector-based vaccine (Oxford/AstraZeneca) [6–8]. Novel pathological concepts, namely vaccine-induced immune thrombotic thrombocytopenia (VITT) [10], vaccine-induced prothrombotic immune thrombocytopenia (VIPIT) [11], and thrombosis with thrombocytopenia syndrome (TTS) [12, 13] were proposed as the underlying mechanisms. Research on thrombosis post vaccination has revealed an association between VITT and the heparin-induced thrombocytopenia (HIT) antibody [7, 14]. Thrombotic events can also be caused by mRNA vaccines, although only a few reports are available and the event frequency after mRNA-based vaccination is lower than after adenovirus vector vaccination [15–18]. In our case, the deceased was found dead 2 days after receiving the mRNA vaccine and her death was suggestive of a vaccine-associated death.

In our case, there were four characteristic macroscopic/microscopic findings: (i) thrombi distributed to most of the systemic organs, especially the heart, (ii) thrombi located primarily in the pericardium-side myocardium and subcapsular regions of the adrenal gland, (iii) thrombi formations 5–40 μm in size found in capillaries and arterioles, and (iv) morphology and IHC staining revealed that the thrombi primarily comprised platelets. Platelet thrombi in the glomerular capillaries and the enlarged subendothelial space of the glomerular basement membrane suggested a vascular endothelial injury in the acute phase. Platelet-rich thrombi are formed by excessive activation of platelet or the vascular endothelial injury and, in our case, the vascular endothelial injury was mainly involved. The absence of thrombi covered with endothelium could be an indication that the thrombi were also in the acute phase. Although laboratory data in the post-mortem investigation were absent, these histopathological findings were compatible with TMA, especially TTP [19–23]; thus, we hypothesised that the underlying pathophysiology in our case was similar to TMA. In this context, TMA meant “histopathological TMA” because TMA is a clinicopathological concept including the triad microangiopathic haemolytic anaemia, thrombocytopenia, and organ dysfunction due to thrombi formation in small vessels. The differential diagnosis of systemic thrombosis involves disseminated intravascular coagulation (DIC), paroxysmal nocturnal haematuria (PNH), autoimmune HIT, TTS/VITT, and TMA. The concept of TMA includes a broad spectrum of diseases [24], such as thrombotic thrombocytopenic purpura (TTP) occurring after adenoviral vector-based vaccination and haemolytic uraemic syndrome (HUS), and TMA with autoimmune conditions, for instance, catastrophic antiphospholipid syndrome (CAPS). Although it is impossible to make a complete differentiation based on each definition and criteria of each disease due to the lack of clinical symptoms, detailed history, and laboratory data including autoantibodies, complement titres, and blood counts, we attempted to histopathologically compare the differentiation of each disease based on the macro/microscopic findings obtained from autopsies and/or the clinically confirmed thrombotic lesion (Table 1). In HUS, thrombi are found primarily in the kidneys [19]. DIC characteristically presents with primarily fibrin thrombi in the renal glomerular capillaries [25, 26], whereas our case presented with platelet thrombi in the afferent/efferent arteriole-level vessels of multiple organs. According to laboratory data including LDH levels one month before the vaccination, the blood count 10 days before the vaccination, and no history or autopsy findings of haematuria, our case seemed incompatible with PNH. Moreover, thrombosis at the hepatic vein is characteristic in PNH [27] and haemosiderin deposition is observed in the renal tubular endothelium in chronic cases [28, 29]. Among the few autopsy case reports that include histopathological analysis of HIT [30–33], the lung and gastrointestinal tract are reported as the main thrombosis sites in HIT. TTS/VITT, which is suspected to be associated with autoimmune HIT, develops 4 to 28 days after vaccination and causes thrombosis characteristically in atypical sites such as the cerebral venous sinus, hepatic vein, and splenic vein [34–40]. Although the thrombi in CAPS share an organ distribution similar to that of our case [41–44], thrombi in CAPS form regardless of the shear stress in arterioles and/or capillaries; thus, it might be difficult to explain our four characteristics findings. As mentioned above, the histopathological findings in our case were different from those of these diseases, although we could not analyse the blood; moreover, the history of trauma and infection that could cause autoimmune HIT are also unknown.

**Table 1:** Review of literature on thrombosis in each thrombotic microangiopathy and relative disease

Pathophysiology	Characteristic thrombi distribution	Chief thrombus vessel	Thrombus components	Other features (if any)	Reference
TTP	Heart, adrenal gland, kidney, pancreas, brain, liver, and spleen	Arteriole/capillary	Platelet	Thrombi were rarely found in the lung, bone marrow, and GI tract	[19–22]
HUS	Kidney and pancreas	Arteriole/capillary	Fibrin		[19]
TMA with catastrophic APS	Kidney, heart, lung, brain, and spleen	Arteriole/capillary	Platelet/Fibrin		[41–44]
TMA with SSc	Kidney (intracapillary)	Arteriole/capillary	Fibrin	Characteristics of vascular endothelial injuries and proliferation on small arterioles	[49, 50]
TMA after bone marrow transplant	Kidney and intestine	Arteriole/small arteries	Fibrin	In the large intestine, the right colon was affected	[51–53]
Pregnancy associated TMA, HELLP	Kidney, lung, and intestine	Arteriole	Platelet/Fibrin	Multiple blackish-reddish patches and confluent hemorrhagic foci on the liver	[54–56]
DIC	Kidney, lung, spleen, and adrenal gland	Arteriole/capillary	Fibrin		[25, 26]
PNH	Hepatic vein	Veins > arteries	Fibrin/platelet	Hemosiderin deposition on the renal tubular epithelium	[27–29]
HIT	Lung, GI tract	Arteries/veins	Platelet		[30–33]
TTS/VITT	CVS, portal, splenic, and SMV	Veins	Platelet/Fibrin		[34–40]
TMA after mRNA-1273 vaccination	Kidney, liver, and GI tract	Small arteries	Platelet	Lupus anticoagulant was positive	[46]
Our case	Heart, adrenal gland, kidney, liver, pancreas, brain, and pons	Arteriole/capillary	Platelet	Thrombi were not found in the lungs or GI tract	

TMA = thrombotic microangiopathy, TTP = thrombotic thrombocytopenic purpura, HUS = haemolytic uraemic syndrome, APS = antiphospholipid syndrome, SSc = systemic sclerosis, HELLP = haemolysis, elevated liver function tests, low platelets syndrome, DIC = disseminated intravascular coagulation, PNH = paroximal nocturnal haemoglobinuria, HIT = heparin-induced thrombocytopenia, TTS = thrombosis with thrombocytopenia syndrome, VITT = vaccine-induced immune thrombotic thrombocytopenia, CVS = cerebral venous sinus, GI = gastrointestinal, SMV = superior mesenteric vein

TMA-like pathophysiology possibly caused severe consumption thrombocytopenia, resulting in multiple petechial haemorrhages in many organs, such as the pancreas and oesophagus, as observed in our case. However, since blood tests were not available at the time of autopsy, it is difficult to determine whether the vaccination caused or triggered her condition resulting in death. In contrast to a number of post-mortem investigations of fatalities following mRNA vaccination that showed myocardial infarction or myocarditis, our histopathological investigation indicated that multiple fatal thromboses following mRNA-based vaccination may have occurred. Macroscopic haemorrhage was also noted on autopsy, consistent with the region in which the deceased complained of pain. Clinicians and researchers should bear in mind that thrombosis may also be one post-mRNA-based vaccination reaction.

Several potential mechanisms that cause vaccine-induced adverse effects have been proposed. For example, TTS/VITT after adenoviral vector-based vaccination is considered to have the same pathophysiology via the anti-platelet factor 4 antibodies [14]. On the other hand, the potential mechanisms of thrombosis after mRNA vaccination remain unclear. One possible mechanism proposed by Trougakos et al. is the spike hypothesis, which states that spike glycoprotein induced by vaccination acts as if it were the spike glycoprotein produced during SARS-CoV-2 infection [45]. In an autopsy case report of TMA after mRNA-1237 vaccination, the author indicated the histopathological similarities of the autopsied case to microvascular injuries of COVID-19 via a complement pathway [46]. On the other hand, cardiac histopathology in lethal cases of COVID-19 reveals haemorrhage in the pericardium, congested microvasculature with/without microhaemorrhage, and hyaline thrombi [47], which are also consistent with our case findings. Considering that the same histopathological changes were observed after viral infection and vaccination, these changes might be related to the common denominator between viral infection and vaccination, namely, the SARS-CoV-2 spike protein and antibodies elicited against it.

TMA-like systemic thrombosis after vaccination itself is a very rare reaction and each case report only indicated temporal relationships; thus, the cause-effect relationship is still unclear. The risk of COVID-19 worsening is thought to be higher than the risk of adverse effects after vaccination. In contrast, a report comparing thrombotic events after influenza vaccination to those after SARS-CoV-2 vaccination showed a significant increase in thrombotic events after COVID-19 vaccination [48]. More rigorous follow-up after vaccination is needed and it is important to detect symptomatic cases early on to allow for early treatment.

In conclusion, this is a rare report of a post-mortem case showing TMA-like multiple systemic thrombi post BNT162b2 mRNA vaccination with a detailed histopathological analysis. Macroscopically, characteristic myocardial haemorrhage was observed, and the histopathology of the characteristic thrombi distribution was similar to that of TMA. Although rare, it is important to keep in mind that fatal adverse reactions may occur after vaccination, and careful follow-up is important after vaccination.

## Electronic supplementary material

Below is the link to the electronic supplementary material.

Supplementary Material 1: Macroscopic haemorrhage of the spleen and oesophagus.tif. a: macroscopic haemorrhage on the surface of the spleen. b: macroscopic haemorrhage on the mucosal surface of the oesophagus

Supplementary Material 2: Vacuolation and anti-C4d-positive cardiomyocytes.tif. a: Vacuolation of the cardiomyocytes. b: anti-C4d immunohistochemistry-positive cardiomyocytes. The positivity is compatible with the vacuolation

Supplementary Material 3: Ultrastructural analysis of microthrombi in the heart.tif. a to d: Microthrombi scanned by transmission electron microscopy. The nuclei of vascular endothelial cells and red blood cells are visible, and platelets and fibrin are found as the boundary indistinct area around the red blood cells with low electron density. The degree of occlusion varies; however, almost all thrombi are non-occlusive. The yellow arrowheads indicate the nuclei of the endothelium, the arrows indicate platelets, the stars indicate fibrin, and the red arrowheads indicate the erythrocytes

## Data Availability

The datasets obtained and analysed in the current study are available from the corresponding author upon reasonable request.

## List of abbreviations

SARS-CoV-2: severe acute respiratory syndrome coronavirus 2.
COVID-19: coronavirus disease 2019.
mRNA: messenger ribonucleic acid.
TMA: thrombotic microangiopathy
IHC: immunohistochemical.
vWF: von Willebrand factor.
LFB: luxol fast blue
ADAMTS13: a disintegrin-like and metalloproteinase with thrombospondin type 1 motifs, member 13.
VITT: vaccine-induced immune thrombotic thrombocytopenia.
VIPIT: vaccine-induced prothrombotic immune thrombocytopenia.
TTS: thrombosis with thrombocytopenia syndrome.
HIT: heparin-induced thrombocytopenia.
TTP: thrombotic thrombocytopenic purpura.
HUS: haemolytic uraemic syndrome.
DIC: disseminated intravascular coagulation.
PNH: paroxysmal nocturnal haematuria.
CAPS: catastrophic antiphospholipid syndrome.

## References

[1] Cucinotta D, Vanelli M. WHO declares COVID-19 a pandemic. Acta Biomed. 2020;157–60. 10.23750/abm.v91i1.9397 PMC7569573 32191675

[2] Ko JY, Danielson ML, Town M, Derado G, Greenlund KJ, Kirley PD (2021). Risk factors for coronavirus disease 2019 (COVID-19)-associated hospitalization: COVID-19-associated hospitalization surveillance network and behavioral risk factor surveillance system. Clin Infect Dis.

[3] Rosner CM, Genovese L, Tehrani BN, Atkins M, Bakhshi H, Chaudhri S, et al. Myocarditis temporally associated with COVID-19 vaccination. Circulation. 2021;502–5. 10.1161/CIRCULATIONAHA.121.055891 PMC8340723 34133885

[4] Larson KF, Ammirati E, Adler ED, Cooper LT, Hong KN, Saponara G (2021). Myocarditis after BNT162b2 and mRNA-1273 vaccination. Circulation.

[5] Abu Mouch S, Roguin A, Hellou E, Ishai A, Shoshan U, Mahamid L (2021). Myocarditis following COVID-19 mRNA vaccination. Vaccine.

[6] Greinacher A, Thiele T, Warkentin TE, Weisser K, Kyrle PA, Eichinger S (2021). Thrombotic thrombocytopenia after ChAdOx1 nCov-19 vaccination. N Engl J Med.

[7] Schultz NH, Sørvoll IH, Michelsen AE, Munthe LA, Lund-Johansen F, Ahlen MT (2021). Thrombosis and thrombocytopenia after ChAdOx1 nCoV-19 vaccination. N Engl J Med.

[8] Scully M, Singh D, Lown R, Poles A, Solomon T, Levi M (2021). Pathologic antibodies to platelet factor 4 after ChAdOx1 nCoV-19 vaccination. N Engl J Med.

[9] Sessa F, Salerno M, Esposito M, Di Nunno N, Zamboni P, Pomara C. Autopsy findings and causality relationship between death and COVID-19 vaccination: a systematic review. J Clin Med. 2021;10. 10.3390/jcm10245876 PMC8709364 34945172

[10] Cines DB, Bussel JB (2021). SARS-CoV-2 vaccine–induced immune thrombotic thrombocytopenia. N Engl J Med.

[11] Xie C, Vincent L, Chadwick A, Peschl H (2021). COVID-19 vaccine induced prothrombotic immune thrombocytopenia. Eur Heart J.

[12] Long B, Bridwell R, Gottlieb M (2021). Thrombosis with thrombocytopenia syndrome associated with COVID-19 vaccines. Am J Emerg Med.

[13] MacNeil JR, Su JR, Broder KR, Guh AY, Gargano JW, Wallace M (2021). Updated recommendations from the advisory committee on immunization practices for use of the Janssen (Johnson & Johnson) COVID-19 vaccine after reports of thrombosis with thrombocytopenia syndrome among vaccine recipients — United States, April 2021. MMWR Recomm Reports.

[14] Baker AT, Boyd RJ, Sarkar D, Teijeira-Crespo A, Chan CK, Bates E (2021). ChAdOx1 interacts with CAR and PF4 with implications for thrombosis with thrombocytopenia syndrome. Sci Adv.

[15] Novak N, Tordesillas L, Cabanillas B (2021). Adverse rare events to vaccines for COVID-19: From hypersensitivity reactions to thrombosis and thrombocytopenia. Int Rev Immunol.

[16] Cari L, Fiore P, Naghavi Alhosseini M, Sava G, Nocentini G (2021). Blood clots and bleeding events following BNT162b2 and ChAdOx1 nCoV-19 vaccine: An analysis of European data. J Autoimmun.

[17] Chen P-W, Tsai Z-Y, Chao T-H, Li Y-H, Hou CJ-Y, Liu P-Y (2021). Addressing vaccine-induced immune thrombotic thrombocytopenia (VITT) following COVID-19 vaccination: a mini-review of practical strategies. Acta Cardiol Sin.

[18] Schulz JB, Berlit P, Diener HC, Gerloff C, Greinacher A, Klein C (2021). COVID-19 vaccine-associated cerebral venous thrombosis in Germany. Ann Neurol.

[19] Hosler GA, Cusumano AM, Hutchins GM (2003). Thrombotic thrombocytopenic purpura and hemolytic uremic syndrome are distinct pathologic entities. A review of 56 autopsy cases. Arch Pathol Lab Med.

[20] Neame PB, Lechago J, Ling ET, Koval A (1973). Thrombotic thrombocytopenic purpura: report of a case with disseminated intravascular platelet aggregation. Blood.

[21] Kwaan HC (1987). Clinicopathologic features of thrombotic thrombocytopenic purpura. Semin Hematol.

[22] Asada Y, Sumiyoshi A, Hayashi T, Suzumiya J, Kaketani K (1985). Immunohistochemistry of vascular lesion in thrombotic thrombocytopenic purpura, with special reference to factor VIII related antigen. Thromb Res.

[23] Tasaki T, Yamada S, Nabeshima A, Noguchi H, Nawata A, Hisaoka M (2015). An autopsy case of myocardial infarction due to idiopathic thrombotic thrombocytopenic purpura. Diagn Pathol.

[24] Brocklebank V, Wood KM, Kavanagh D (2018). Thrombotic microangiopathy and the kidney. Clin J Am Soc Nephrol.

[25] Watanabe T, Imamura T, Nakagaki K, Tanaka K (1979). Disseminated intravascular coagulation in autopsy cases its incidence and clinicopathologic significance. Pathol Res Pract Gustav Fischer Verlag.

[26] Ohashi R, Ishii H, Naito Z, Shimizu A (2014). Morphological spectrum of renal pathology and its correlation to clinical features in patients with disseminated intravascular coagulation: a study involving a series of 21 autopsy cases. Pathol Int.

[27] Ziakas PD, Poulou LS, Rokas GI, Bartzoudis D, Voulgarelis M (2007). Thrombosis in paroxysmal nocturnal hemoglobinuria: sites, risks, outcome. An overview. J Thromb Haemost.

[28] Chow KM, Lai FM, Wang AY, Chan YL, Tang NL, Li PK (2001). Reversible renal failure in paroxysmal nocturnal hemoglobinuria. Am J Kidney Dis.

[29] Puri V, Gandhi A, Sharma S (2017). Renal biopsy in paroxysmal nocturnal hemoglobinuria: an insight into the spectrum of morphologic changes. Indian J Nephrol.

[30] Ichinoe M, Mikami T, Ujiie S, Suzuki K, Okayasu I (2009). Heparin-induced thrombocytopenia with multiple organized thrombi accompanied by unusual cholesterin deposition: Autopsy case after long-term follow up: Case Report. Pathol Int.

[31] Sawaki D, Otani Y, Kobayakawa N, Sekita G, Fukushima K, Takeuchi H (2004). A case of heparin-induced thrombocytopenia with sepsis and congestive heart failure first autopsy report on Japan. Circ J.

[32] Hermanns B, Janssens U, Handt S, Füzesi L (1998). Pathomorphological aspects of heparin-induced thrombocytopenia II (HIT-II syndrome). Virchows Arch.

[33] Theuerkauf I, Lickfett L, Harbrecht U, Pohl C, Fischer H, Pfeifer U (2000). Segmental hepatic vein thrombosis associated with heparin-induced thrombocytopenia II. Virchows Arch.

[34] Althaus K, Möller P, Uzun G, Singh A, Beck A, Bettag M (2021). Antibody-mediated procoagulant platelets in SARS-CoV-2-vaccination associated immune thrombotic thrombocytopenia. Haematologica.

[35] Wiedmann M, Skattør T, Stray-Pedersen A, Romundstad L, Antal E-A, Marthinsen PB (2021). Vaccine induced immune thrombotic thrombocytopenia causing a severe form of cerebral venous thrombosis with high fatality rate: a case series. Front Neurol.

[36] Pomara C, Sessa F, Ciaccio M, Dieli F, Esposito M, Garozzo SF (2021). Post-mortem findings in vaccine-induced thrombotic thombocytopenia. Haematologica.

[37] Roncati L, Manenti A, Corsi L (2022). A three-case series of thrombotic deaths in patients over 50 with comorbidities temporally after modRNA COVID-19 vaccination.

[38] Pomara C, Salerno M, Esposito M, Sessa F, Certo F, Tripodo C (2022). Histological and immunohistochemical findings in a fatal case of thrombotic thrombocytopenia after ChAdOx1 nCov-19 vaccination. Pathol Res Pract.

[39] Fanni D, Saba L, Demontis R, Gerosa C, Chighine A, Nioi M (2021). Vaccine-induced severe thrombotic thrombocytopenia following COVID-19 vaccination: A report of an autoptic case and review of the literature. Eur Rev Med Pharmacol Sci.

[40] Mele F, Tafuri S, Stefanizzi P, Amati D, Calvano A, Leonardelli M. M, et al. Cerebral venous sinus thrombosis after COVID-19 vaccination and congenital deficiency of coagulation factors: Is there a correlation? Hum Vaccin Immunother. 2022;2095166. 10.1080/21645515.2022.2095166 PMC9746424 35895937

[41] Bucciarelli S, Cervera R, Espinosa G, Gómez-Puerta JA, Ramos-Casals M, Font J (2006). Mortality in the catastrophic antiphospholipid syndrome: Causes of death and prognostic factors. Autoimmun Rev.

[42] Bucciarelli S, Espinosa G, Cervera R, Erkan D, Gómez-Puerta JA, Ramos-Casals M (2006). Mortality in the catastrophic antiphospholipid syndrome: causes of death and prognostic factors in a series of 250 patients. Arthritis Rheum.

[43] Mizuno R, Fujimoto S, Fujimoto T, Nishino T, Shiiki H, Hashimoto T (2000). Catastrophic antiphospholipid antibody syndrome in systemic lupus erythematosus: An autopsy case report of a young woman. Intern Med.

[44] Kinjo K, Terabe H, Ogawa M, Takeda H, Cho D, Hoshino S (2008). A case of catastrophic antiphospholipid syndrome presenting with acute respiratory distress syndrome as the initial manifestation. Rheumatol Int.

[45] Trougakos IP, Terpos E, Alexopoulos H, Politou M, Paraskevis D, Scorilas A (2022). Adverse effects of COVID-19 mRNA vaccines: the spike hypothesis. Trends Mol Med.

[46] Kamura Y, Terao T, Akao S, Kono Y, Honma K, Matsue K (2022). Fatal thrombotic microangiopathy with rhabdomyolysis as an initial symptom after the first dose of mRNA–1273 vaccine: A case report. Int J Infect Dis.

[47] Haslbauer JD, Tzankov A, Mertz KD, Schwab N, Nienhold R, Twerenbold R (2021). Characterisation of cardiac pathology in 23 autopsies of lethal COVID-19. J Pathol Clin Res.

[48] Vallone MG, Falcón AL, Castro HM, Ferraris A, Cantarella RF, Staneloni MI, et al. Thrombotic events following Covid-19 vaccines compared to Influenza vaccines. Eur J Intern Med. 2022. 10.1016/j.ejim.2022.03.002 PMC8904150 35288031

[49] Ishizu A, Fukaya S, Tomaru U, Katsumata K, Suzuki A, Umemoto Y (2012). Acute renal failure due to thrombotic microangiopathy in patient with scleroderma: autopsy case report. Ann Vasc Dis.

[50] Gallan AJ, Chang A (2020). A new paradigm for renal thrombotic microangiopathy. Semin Diagn Pathol.

[51] Yamada R, Nemoto T, Ohashi K, Tonooka A, Horiguchi S ichiro, Motoi T, et al. Distribution of transplantation-associated thrombotic microangiopathy (TA-TMA) and comparison between renal TA-TMA and intestinal TA-TMA: autopsy study. Biol Blood Marrow Transplant. 2020;26:178–88. 10.1016/j.bbmt.2019.08.025 31491486

[52] Siami K, Kojouri K, Swisher KK, Selby GB, George JN, Laszik ZG (2008). Thrombotic microangiopathy after allogeneic hematopoietic stem cell transplantation: An autopsy study. Transplantation.

[53] Goyama S, Takeuchi K, Kanda Y, Nannya Y, Chiba S, Fukayama M (2012). Post-transplant endothelial disorder after hematopoietic SCT: A blinded autopsy study. Bone Marrow Transplant.

[54] Tsokos M, Longauer F, Kardosová V, Gavel A, Anders S, Schulz F (2002). Maternal death in pregnancy from HELLP syndrome. A report of three medico-legal autopsy cases with special reference to distinctive histopathological alterations. Int J Legal Med.

[55] Zununi Vahed S, Rahbar Saadat Y, Ardalan M (2021). Thrombotic microangiopathy during pregnancy. Microvasc Res.

[56] Tsokos M, Tsokos M (2004). Pathological features of maternal death from HELLP syndrome. Forensic Pathology Reviews.

