# Supplementary figures and images for: Histopathologically TMA-like distribution of multiple organ thromboses following the initial dose of the BNT162b2 mRNA vaccine (Comirnaty, Pfizer/BioNTech): an autopsy case report
Source: Thromb J. 2022 Oct 6;20:61. doi: 10.1186/s12959-022-00418-7 (PMC9540301; doi:10.1186/s12959-022-00418-7)

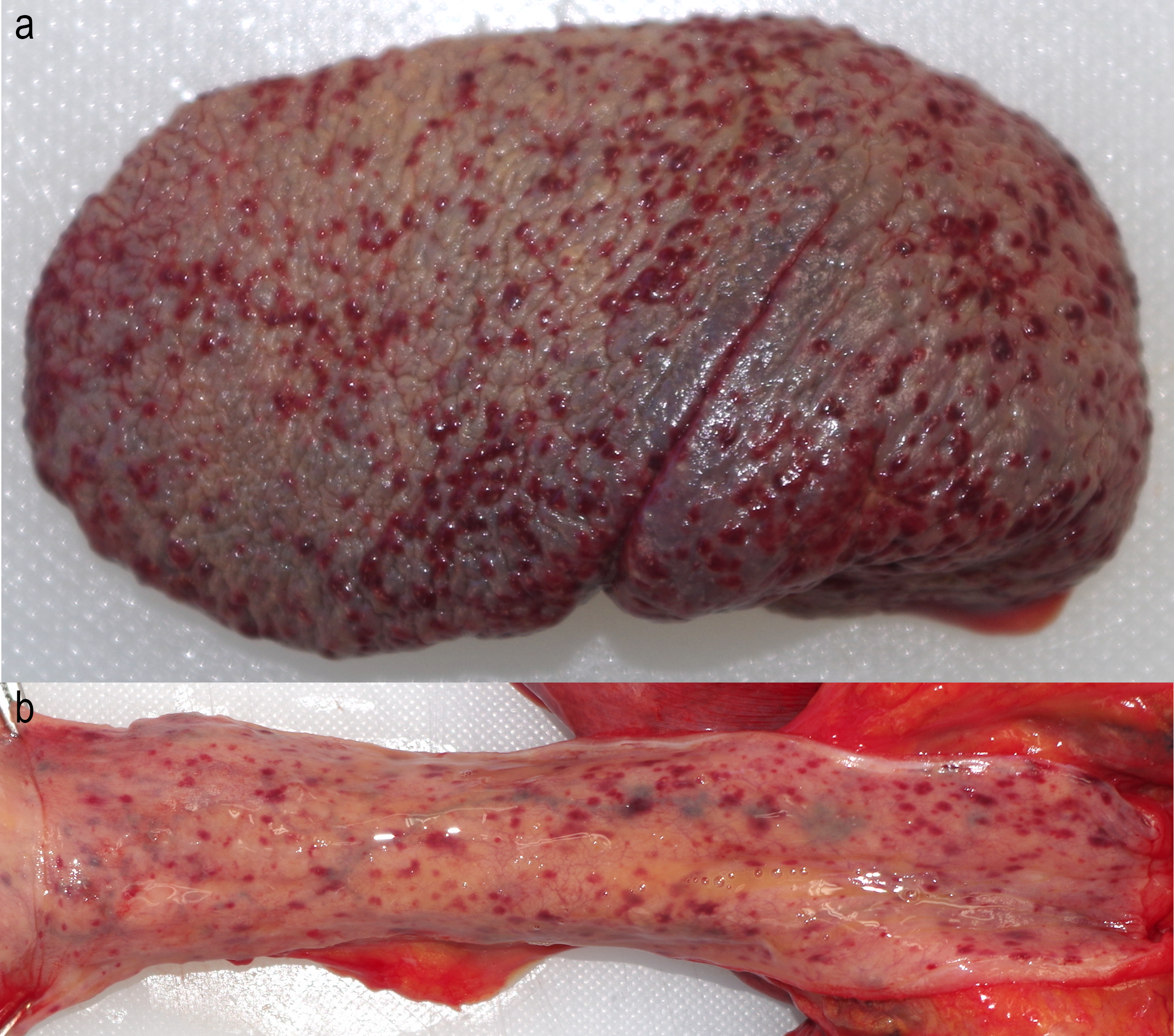

Supplement: Supplementary file 1 — Supplementary Material 1: Macroscopic haemorrhage of the spleen and oesophagus.tif. a: macroscopic haemorrhage on the surface of the spleen. b: macroscopic haemorrhage on the mucosal surface of the oesophagus [file 12959_2022_418_MOESM1_ESM.tif]

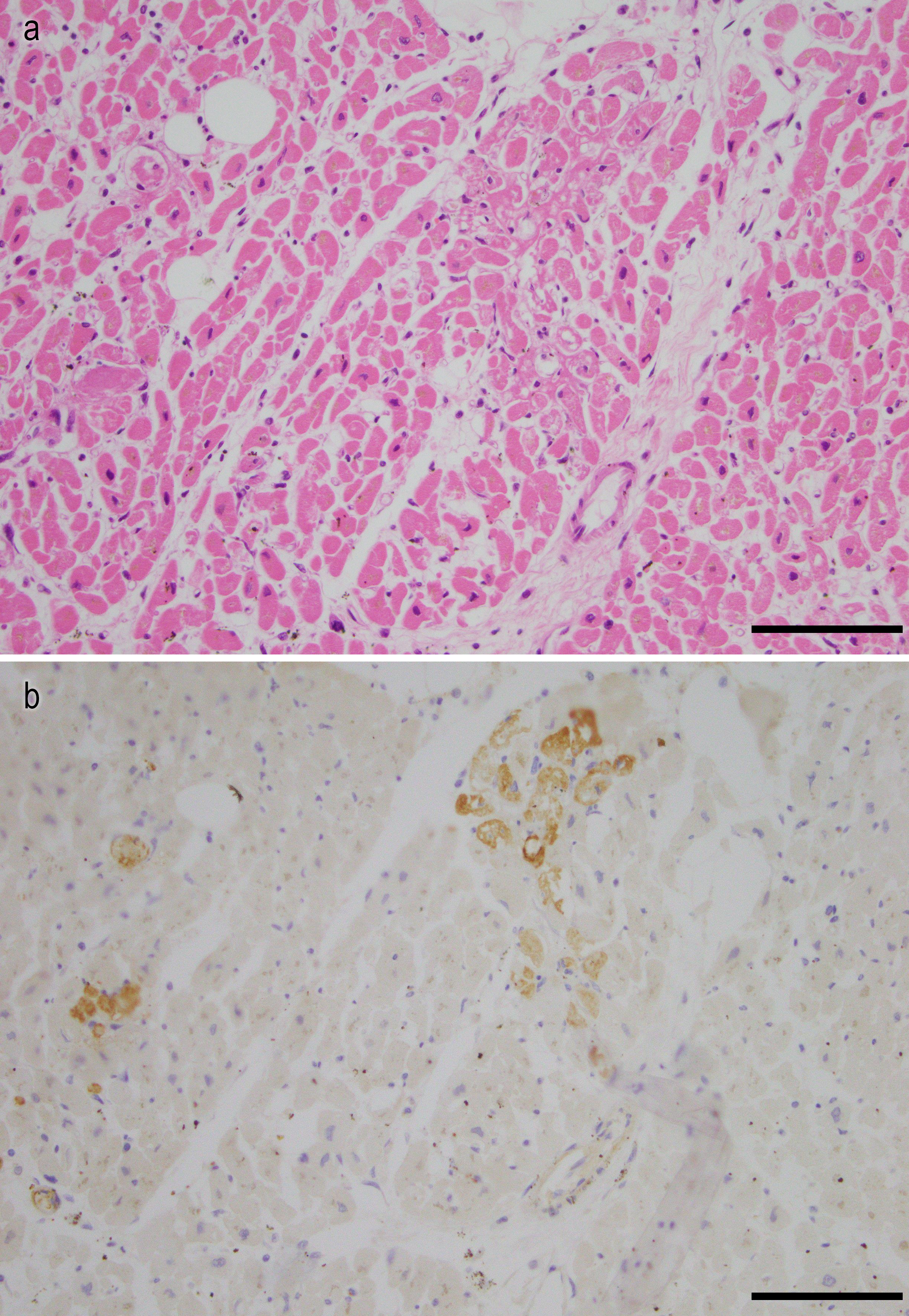

Supplement: Supplementary file 2 — Supplementary Material 2: Vacuolation and anti-C4d-positive cardiomyocytes.tif. a: Vacuolation of the cardiomyocytes. b: anti-C4d immunohistochemistry-positive cardiomyocytes. The positivity is compatible with the vacuolation [file 12959_2022_418_MOESM2_ESM.tif]

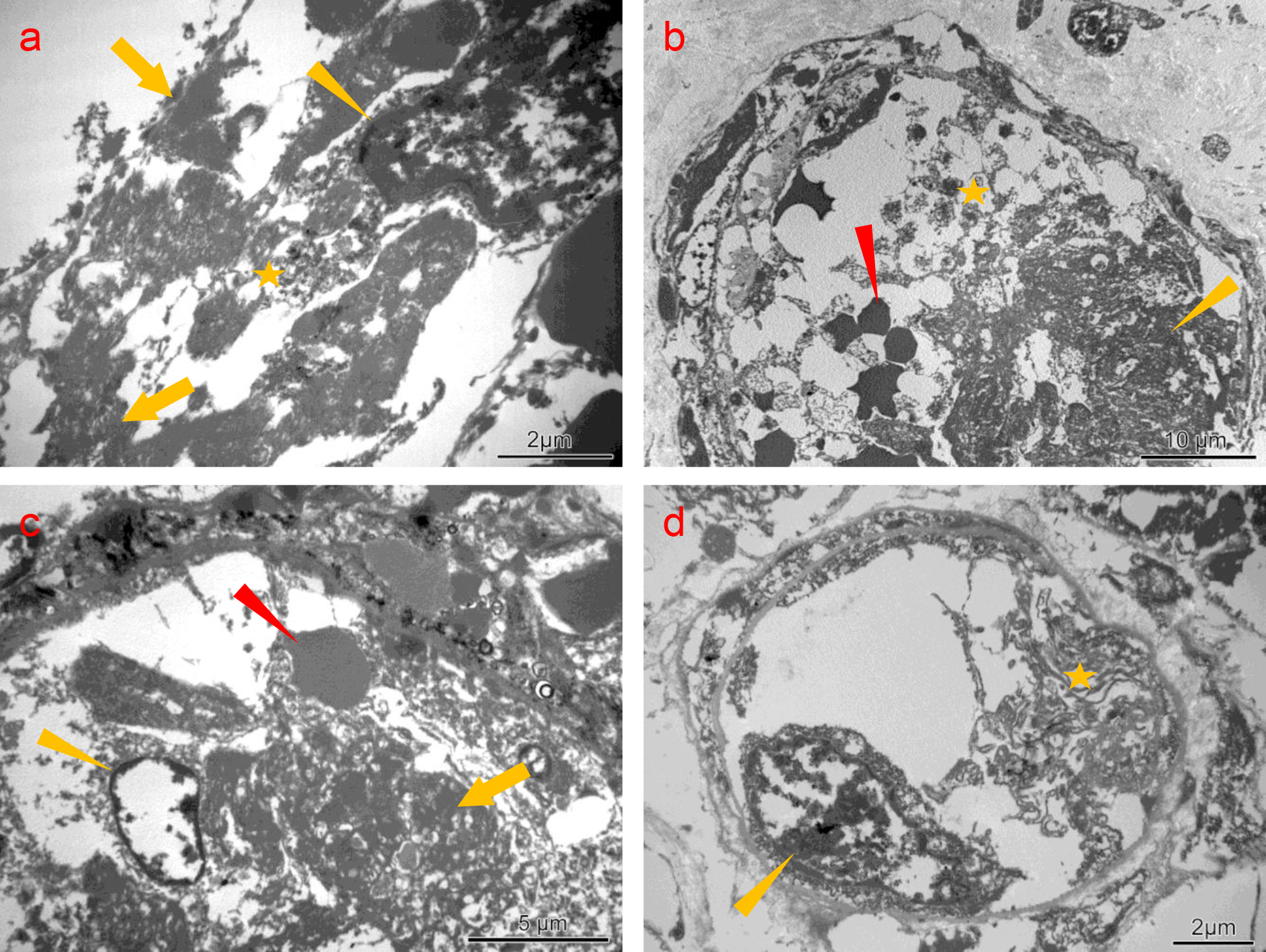

Supplement: Supplementary file 3 — Supplementary Material 3: Ultrastructural analysis of microthrombi in the heart.tif. a to d: Microthrombi scanned by transmission electron microscopy. The nuclei of vascular endothelial cells and red blood cells are visible, and platelets and fibrin are found as the boundary indistinct area around the red blood cells with low electron density. The degree of occlusion varies; however, almost all thrombi are non-occlusive. The yellow arrowheads indicate the nuclei of the endothelium, the arrows indicate platelets, the stars indicate fibrin, and the red arrowheads indicate the erythrocytes [file 12959_2022_418_MOESM3_ESM.tif]
